# Methods for characterizing pollen fitness in *Cannabis sativa* L.

**DOI:** 10.1371/journal.pone.0270799

**Published:** 2022-07-07

**Authors:** Sydney B. Wizenberg, Michelle Dang, Lesley G. Campbell

**Affiliations:** Department of Chemistry and Biology, Toronto Metropolitan University (recently renamed), Toronto, Ontario, Canada; USDA-ARS Southern Regional Research Center, UNITED STATES

## Abstract

Pollen grains are male gametophytes, an ephemeral haploid generation of plants, that commonly engage in competition for a limited supply of ovules. Since variation in reproductive capabilities among male gametophytes may influence the direction and pace of evolution in populations, we must be able to quantify the relative fitness of gametophytes from different sires. To explore this, we estimated the relative fitness of groups of male gametophytes in a dioecious, wind-pollinated model system, *Cannabis sativa*, by characterizing the non-abortion rate (measured via chemical staining) and viability (measured via *in vitro* germination) of pollen from multiple sires. Pollen viability quickly declined within two weeks of anther dehiscence, and pollen stored under freezer conditions did not germinate regardless of storage time. In contrast, pollen non-abortion rates declined slowly and persisted longer than the lifetime of a sporophyte plant under both room temperature and freezer conditions. Pollen samples that underwent both viability and non-abortion rate analysis displayed no significant correlation, implying that researchers cannot predict pollen viability from non-abortion rates, nor infer male gametophytic fitness from a single measure. Our work demonstrates two independent, differential approaches to measure proxies of male fitness in *C*. *sativa*.

## Introduction

Pollen grains are male gametophytes, representing an ephemeral, haploid generation in the plant life cycle [1–3]. Pollen compete for opportunities to fertilize ovules and thus, influence differential reproductive success, a key condition for microevolution [4–7]. Post-meiotic gene expression and the abundance of pollen produced by many gymnosperms and angiosperms sets the stage for intense competition among pollen-derived sperm cells for the often limited supply of ovules, wherein sporophyte reproductive success relies on not only successful dispersal of pollen, but also successful pollination of stigmas and subsequent pollen germination and ovule fertilization [6, 7]. To better understand and explore the competition that might occur at the stage of pollen production and release, we must be capable of characterizing and estimating their relative fitness, including: abortion rates and viability [8]. Pollen abortion rates, quantified using chemical staining, measures cytoplasmic degradation of the regenerative cell to quantify the proportion of pollen from a sire that contain intact regenerative nuclei [9–21]. Pollen abortion rates measure how many pollen grains are capable of engaging in reproduction under any condition, as a degraded cytoplasm indicates senescence and death of the gamete, as opposed to measuring how many pollen grains may germinate under a particular set of conditions. Comparatively, pollen viability is quantified through measurement of the proportion of pollen grains that germinate under standardized conditions [22–30]. Successful germination of a pollen grain requires rehydration of the apertures and subsequent protrusion of the pollen tube towards the ovule, following which the pollen tube transports the male gamete to the female ovule to produce a zygote [23, 31]. Both pollen viability and non-abortion rates are measures of the proportion of male gametophytes within a sample that is capable of engaging in reproduction under standardized conditions, and quantification of these measures could provide insight into why, or how, some male plants confer a reproductive advantage.

Pollen abortion rates can be tested using many methods [11, 13, 17, 32], but one common means of differential staining that works on many species is the Alexander stain. Originally published in 1969, the Alexander stain uses acid fuchsin and malachite green to test if the cytoplasm containing the regenerative nucleus is intact [10]. Malachite green stains the exine and intine cell walls blue, while acid fuchsin is absorbed by the cytoplasm, resulting in a pink stain [10]. Aborted and non-aborted pollen grains are differentiated by the resulting coloration, wherein pollen that stains pink within the vegetative or regenerative cell do not contain an in-tact regenerative nucleus, and are therefore incapable of fertilizing an ovule regardless of the external germination conditions [10]. Originally containing chloral hydrate, mercuric chloride, and phenol, a simplified version of the Alexander stain was developed and tested in 2010, allowing wider applications of this method due to the removal of some toxic and difficult to acquire chemical components [33]. This simplified method was successful at differentiating between aborted and non-aborted pollen in numerous test species (*Gingko biloba*, *Pinus resinosa*, *Acer rubrum*, *Arabidopsis thaliana*, *Betula populifolia*, *Fragaria versca*, *Lonicera tatarica*, *Oryza sativa*, *Prunus padus*, *Rhododendron mucronulatum*) and shows promise in its ability to act as one standardized, interspecific method of estimating pollen abortion rates [33]. Measuring pollen viability through *in vitro* germination is more challenging to standardize, as germination medias are typically developed for individual species, based on their distinct biochemical germination signals [22, 34]. Some components, such as water, sucrose, boric acid, and polyethylene glycol, are used frequently in medias developed for different species, while other growth inducing additives such as calcium chloride, potassium chloride, potassium nitrate, and magnesium sulphate, can vary significantly in their quantity [22, 35–43]. All germination medias must contain a combination of carbohydrate sources in addition to growth inducing additives to mimic biochemical indicators of stigma proximity and induce rehydration and subsequent pollen tube growth [22, 23]. As a result of variation in the composition of germination medias between species, standardization is difficult, and prevents relative comparisons of viability between species that rely on different germination medias.

*Cannabis sativa* L. is a dioecious crop frequently cultivated for its cannabinoids, fibre and seeds [44–50]. This species is anemophilous, and its exine morphology reflects this dispersal strategy, meaning its pollen grains are not ornamented and thus well suited to rapid movement coinciding with any changes in air flow [51, 52]. Industrial facilities that produce high cannabinoid yielding plants often produce sinsemilla, unpollinated floral biomass, because growing female plants in strict isolation of pollen is required to prevent a reduction in cannabinoid content and the length of trichome dense stigmas [53, 54]. In this light, investigating the behaviour and related characteristics of pollen in this economically valuable species could improve our ability to control pollination risk within growing facilities, in addition to answering basic scientific questions about male gametophytes and their life cycle. Previous research investigating pollen viability and abortion rates in *C*. *sativa* has demonstrated that different methods can produce significantly different results. Zottini *et al*. investigated the effect of gamma ray irradiation on pollen viability and abortion rates, using loaded fluorescein diacetate to differentiate between aborted and non-aborted grains under a microscope, and 5 different germination medias [55]. They found that gamma ray irradiation did not affect abortion rates, but did drastically reduce rates of *in vitro* germination, and noted that measures of viability and abortion rates differed from one another [55]. Comparatively, Choudhary *et al*. tested three different measures of viability: Alexander’s stain, triphenyl tetrazolium chloride, and flurochromatic reaction; documenting a substantial decline in abortion rates within three days of anther dehiscence [56]. More recently, Gaudet *et al*. developed a media for *in vitro* germination and investigated long-term cryopreservation of *C*. *sativa* pollen for use in breeding programs [57]. They found that pollen collected at different developmental stages also differed in its germination capabilities; some samples maintained viability past three weeks, while others declined rapidly in the first two weeks of storage [57]. Pollen stored in wheat flour and liquid nitrogen maintained its ability to germinate *in vitro* for up to 4 months, though the germination rates remained low over-all, even for fresh pollen [57]. Noting the inconsistencies in methodologies and conclusions among previous publications on this topic, and building on our previous work [58], we set out to investigate *C*. *sativa* pollen viability and abortion rates, under the broader goal of developing a framework for measuring male gametophytic fitness. Accordingly, our research asks:

1. Do *C*. *sativa* pollen non-abortion rates differ when stored under standard and freezer temperature conditions?
2. Do *C*. *sativa* pollen viability estimates differ when stored under standard and freezer temperature conditions?
3. Can estimates of *C*. *sativa* pollen non-abortion rates predict estimates of pollen viability?

## Materials and methods

Throughout a two year period (2019–2021) we developed and tested a framework for measuring the relative fitness of groups of male gametophytes through two characteristics: pollen viability, and non-abortion rates. We utilized previously established methods, the Alexander stain for measuring pollen non-abortion rates [10, 33], and Gaudet *et al*.’s media for *in vitro* germination [57], and tested them on *C*. *sativa* to establish baseline values of comparison and measure degradation of both characteristics across time. Additionally, we tested if pollen grain viability and non-abortion rates were correlated by assessing both measures on samples from the same pollen source. In all three of the experiments described below we selected early flowering males to minimize variation in pollen characteristics and maintained all experimental conditions and horticultural methods.

### Plant genotype and cultivation methods

To test hypotheses around pollen viability and non-abortion rates, we grew CFX-2 (Hemp Genetics International, Saskatoon, Saskatchewan, Canada), a hemp cultivar of *C*. *sativa* with an expected total tetrahydrocannabinol (THC) content of less than 0.01%. The project was divided into three distinct experiments, the first of which explored long term pollen non-abortion rates, the second of which explored short term pollen non-abortion rates, and the third of which explored pollen viability and the relationship between non-abortion rates and viability. The three experiments started on May 29^th^, 2019; October 21^st^, 2019; and April 2^nd^, 2021, respectively; all using the same horticultural methods. For each experiment, we germinated 20 seeds in a terracotta germination pot (ANVÄNDBAR Sprouter; IKEA, Delft, The Netherlands) for three days, watering once daily with 25 mL of filtered water (Milli-Q purification system #F7KA48180D, Millipore Canada Ltd., Etobicoke, Ont.). Following germination, we planted all 20 seedlings in SC-10 cone-tainers^®^ (Stuewe and Sons Inc., Tangent, Oregon, USA) filled with 200 mL of moistened PRO-MIX mycorrhizae peat moss growing medium (Premier Tech, Riviere-du-Loup, Quebec, Canada). Seven days later we transplanted the seedlings into circular pots (15 cm. diameter x 11 cm. height) filled with approximately 1 L. of moistened PRO-MIX mycorrhizae peat moss growing medium. We then placed the seedlings under 24h lighting from fluorescent T8 bulbs (F32W, Canarm lighting and fans, Brockville, Ontario, Canada) for four weeks, following which we switched to a 12h lighting photoperiod regimen to induce flowering for the remainder of the experiment. We watered each plant twice weekly with 50 mL of filtered water and fertilized them once weekly with 250 mL of 0.4% Miracle-Gro^®^ (10-10-10 NPK; Scotts Miracle-Gro, Marysville, Ohio, USA) diluted in filtered water.

### Pollen collection

Once floral development was initiated, we identified and tagged ten male plants using floral morphology, i.e., the visible development of pollen-producing inflorescences at apical branching junctions. Of the ten male plants that were identified and tagged, we selected the five that flowered early for inclusion in the experiment to minimize any variation in pollen characteristics as a result of phenological differences [57]. The five tagged male plants were closely monitored until the inflorescences began to swell, showing visible protrusion of mature pollen sacs, indicating that anther dehiscence would occur. On the first day of anther dehiscence, we hand collected pollen [58] in 1.7 mL centrifuge tubes (LIFEGENE graduated micro-centrifuge tubes, Modiin, Israel). Pollen samples in centrifuge tubes were left unsealed to dry for 1hr before sealing, following which we stored them under one of two experimental conditions; samples were labelled and stored at either ‘room temperature’ conditions of 22 ± 0.95°C, or ‘freezer’ conditions of -4°C. For each of the first five males that flowered in our population, we collected pollen twice on the initial day of anther dehiscence, with one sample being stored at ‘room temperature’ and the other being stored under ‘freezer’ conditions.

### Quantifying pollen non-abortion rates

**Fig 1:**
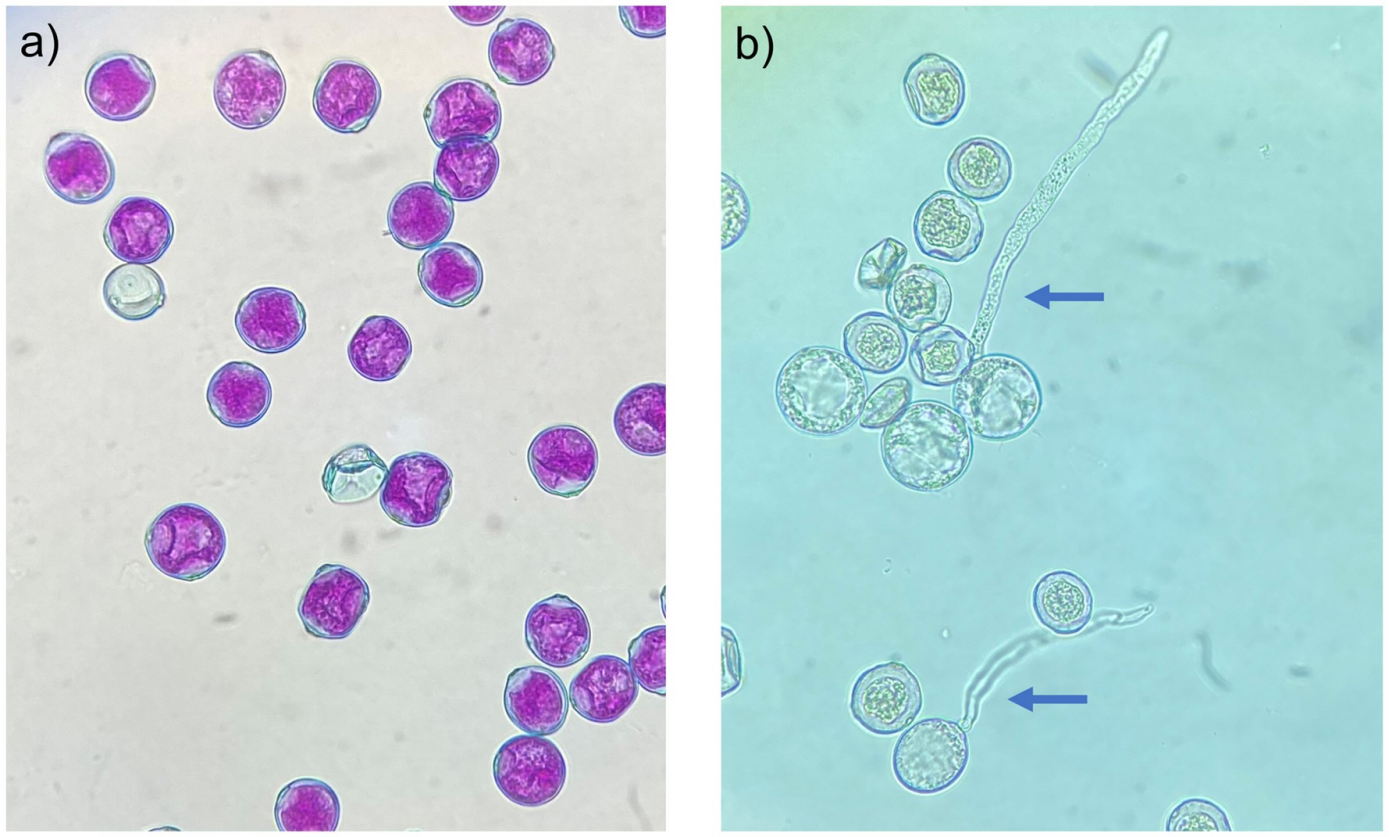
Differential staining and *in vitro* germination of *Cannabis sativa* pollen. (a) Differential staining of non-aborted (pink cytoplasm with blue exines) and aborted pollen grains (blue exines), showing absorption of pink acid fuchsin in the cytoplasm of functional pollen grains. (b) *In vitro* germination of viable and inviable pollen grains, showing protrusion of the pollen tube in viable pollen grains (blue arrows).

To test pollen non-abortion rates, we prepared a batch of the modified Alexander stain [33], and before initiating the experiment, informally tested it on multiple pollen samples to confirm the presence of differential staining for pollen containing a functional cytoplasm (containing an intact regenerative nucleus and gamete) and those containing a degraded cytoplasm (Fig 1a). To assemble the stain, we combined 10 mL of 95% alcohol, 1 mL of diluted malachite green (1% solution in 95% alcohol), 54.5 mL of distilled water, 25 mL of glycerol, 5 mL of diluted acid fuchsin (1% solution in distilled water), 0.5 mL of diluted orange G (1% solution in distilled water), and 4 mL of glacial acetic acid. To evaluate the non-abortion rates of each pollen specimen collected, we unsealed the relevant sample and used a fresh cotton swab (Q-tips, Unilever, London, UK) to apply a small sample of pollen (ranging between 800–10,000 pollen grains) to a 75 mm x 25 mm glass microscope slide. We pipetted 20 μL of the modified Alexander stain directly onto the applied pollen sample and heated the prepared slide 10 cm above a Bunsen burner for 5 s. to allow the stain to set. Once the heated sample had cooled, we applied a glass slide cover (25 mm x 25 mm), and the sample was left to incubate at room temperature (22 ± 0.95°C) for 24 hr. To estimate the proportion of pollen that stained as non-aborted as a percentage of the total sample, we used a light microscope (Zeiss Primo Star Upright Light Microscope, Carl Zeiss Canada Ltd., Toronto, Ont.) to perform vertical transects at 10x magnification (covering the entire length and width of the 2 mm.^2^ slide cover), counting the number of non-aborted grains (stained pink) and the number of aborted grains (stained blue) using two hand-held tally counters (Uline, Pleasant Prairie, Wisconsin, USA). Similarly to previous work [58], we kept a tally of the number of burst pollen grains on each slide that was excluded from totals; they showed an over-all low abundance of <1%. Any pollen grains that formed a clump were excluded from all counts as it was not feasible to differentiate between non-aborted and aborted staining. Under room temperature conditions, we measured pollen non-abortion rates after initial anther dehiscence then weekly for the first four weeks of the experiment, and again after eight and twelve weeks. Under freezer temperature conditions, we measured pollen abortion rates after initial anther dehiscence then in 16-week intervals until 96 weeks, after which we concluded the experiment.

### Quantifying pollen viability

To test pollen viability, we assembled a germination media recipe developed for *C*. *sativa* by Gaudet *et al*. [57] and informally tested it on multiple pollen samples to confirm that it induced germination and that germinated and non-germinated pollen were differentiable under a light microscope (Fig 1b). To assemble the media, we combined 10% sucrose, 0.005% H_3_BO_3_, 10 mM CaCl_2_, 0.05 mM Kh_2_PO_4_, and 6% PEG 4000, then diluted it in 250 mL of distilled water. We unsealed each relevant sample and used a fresh cotton swab (Q-tips, Unilever, London, UK) to apply a small sample of pollen (800–10,000 pollen grains) to a 75 mm x 25 mm glass microscope slide. We heated 10 mL of the prepared germination media in a 40ml glass beaker placed on a hot plate at 70°C for 10 min, at which point it reached a temperature of 35°C, then we pipetted 50 μL of the heated media onto the pollen sample and immediately sealed it with a glass slide cover (25 mm x 25 mm). We incubated the media at 28°C (Fisher Scientific 6845 Isotemp Incubator 650D, Waltham, Massachusetts, USA) for 24 hr in a sealed petri dish (8.5 cm x 8.5 cm x 1 cm) containing a filter paper (11 cm x 21 cm) soaked in 10 mL of water to increase the relative humidity during incubation. After 24 hr, germination was visible (Fig 1b) and we estimated the proportion of viable pollen grains as a percentile of the total pollen sample using a light microscope. We performed vertical transects at 10x magnification (covering the entire length and width of the 22 mm.^2^ slide cover), counting the number of germinated grains (rehydrated and containing a protruding pollen tube) and the number of non-germinated grains using two hand-held tally counters. Similarly to previous work [58], we kept a tally of the number of burst pollen grains on each slide that was excluded from totals; they showed an over-all low abundance of <1%. Any pollen grains that formed a clump were excluded from all counts as it was not feasible to differentiate between germinated and non-germinated pollen. Under both room temperature and freezer conditions, we measured pollen viability after initial anther dehiscence then weekly until all samples showed no *in vitro* germination, at which point viability was deemed to be zero.

### Statistical analyses

We conducted all analyses in R v.4.0.2, using the *stats* package (2019-04-06, R Core Team, 2019). We evaluated variable distributions using residual QQ plots and all models were deemed to be sufficiently parametric. To determine if data collected conformed to typical patterns of pollen survival curves, we created linear models using the ‘lm()’ function. Weeks post anther dehiscence was the experimental factor used to predict an outcome of either pollen non-abortion rate (%), the proportion of pollen grains that maintained an intact regenerative nuclei, or viability (%), the proportion of pollen grains that germinated *in vitro*. We separated data sets based on storage conditions, and each was analyzed as an independent experiment. We gauged model strength through the adjusted R^2^, a goodness-of-fit statistics adjusted for the number of observations, and the residual standard error, both of which were reported using the ‘summary()’ function on each respective linear model. Once we had determined that the data represented typical pollen survival behavior, an additional data set which contained paired estimates of pollen non-abortion rates and viability (from the same parent pollen sample) underwent analysis to determine if any relationship existed between the two measures of male gametophytic fitness. Though originally we utilized a general linear model to investigate this relationship, the related summary statistics implied no dependent relationship (reported in the results), resulting in an insufficient model fit and weak predictive power. We attempted transforming the data to investigate an inverse exponential relationship but this worsened the model fit and decreased the models predictive power. Bearing this in mind, we opted to investigate any potential non-parametric relationship by performing the Wilcoxon signed-rank test, using the ‘wilcox.test()’ function contained in the *stats* package.

## Results

**Fig 2:**
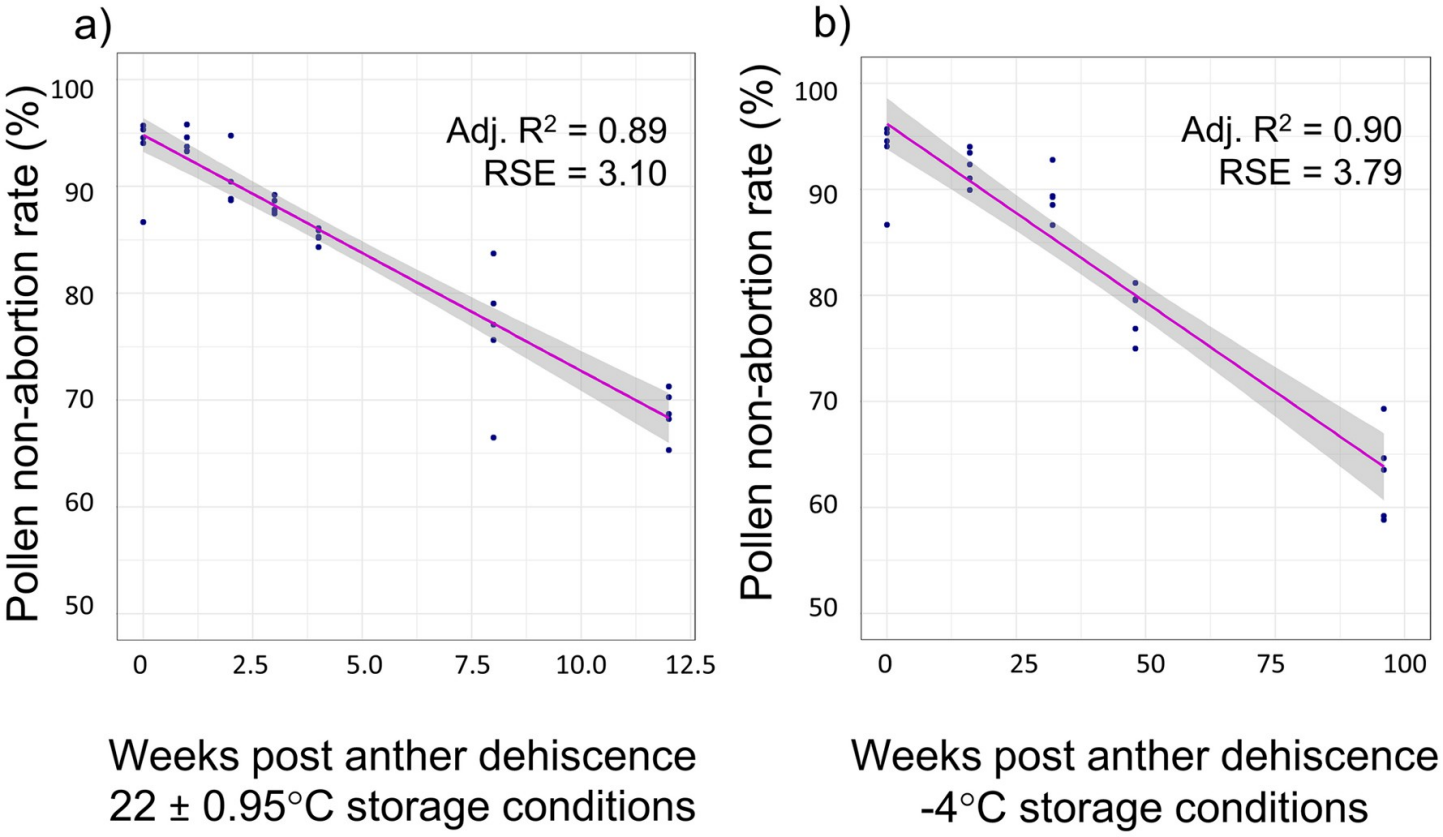
Pollen grain non-abortion rates under two experimental conditions. The linear relationship between weeks post anther dehiscence and the non-abortion rate of pollen stored under (a) room temperature conditions (22 ± 0.95°C) and (b) freezer temperature conditions (-4°C).

Pollen viability and non-abortion rates were generally high on the first day they were measured and declined with time. Samples tested directly after collection had an average proportion of non-aborted pollen of 93.35% (± 3.74%) and an average viability of 38.69% (± 3.15%). Pollen stored under room temperature conditions (22 ± 0.95°C) showed a consistent increase in abortion rates over the 12-week monitoring program, and the associated linear model predicted that pollen samples under these conditions may maintain in-tact regenerative nuclei for up to 39 weeks, approximately nine months post anther dehiscence. The linear model fitted to predict pollen non-abortion rates when stored under room temperature conditions showed a strong model fit (Adj. R^2^ = 0.89, RSE = 3.12, df = 33, F = 277, p < 0.001; Fig 2a). Pollen stored under freezer conditions (-4°C) maintained high non-abortion rates even after 96 weeks of storage, and the associated linear model predicted that pollen stored under freezer conditions may maintain some intact regenerative nuclei up to 261.5 weeks, approximately five years after anther dehiscence. The linear model fitted to predict pollen non-abortion rates when stored in a freezer also showed a strong model fit (Adj. R^2^ = 0.89, RSE = 3.79, df = 23, F = 214.8, p < 0.001; Fig 2b).

**Fig 3:**
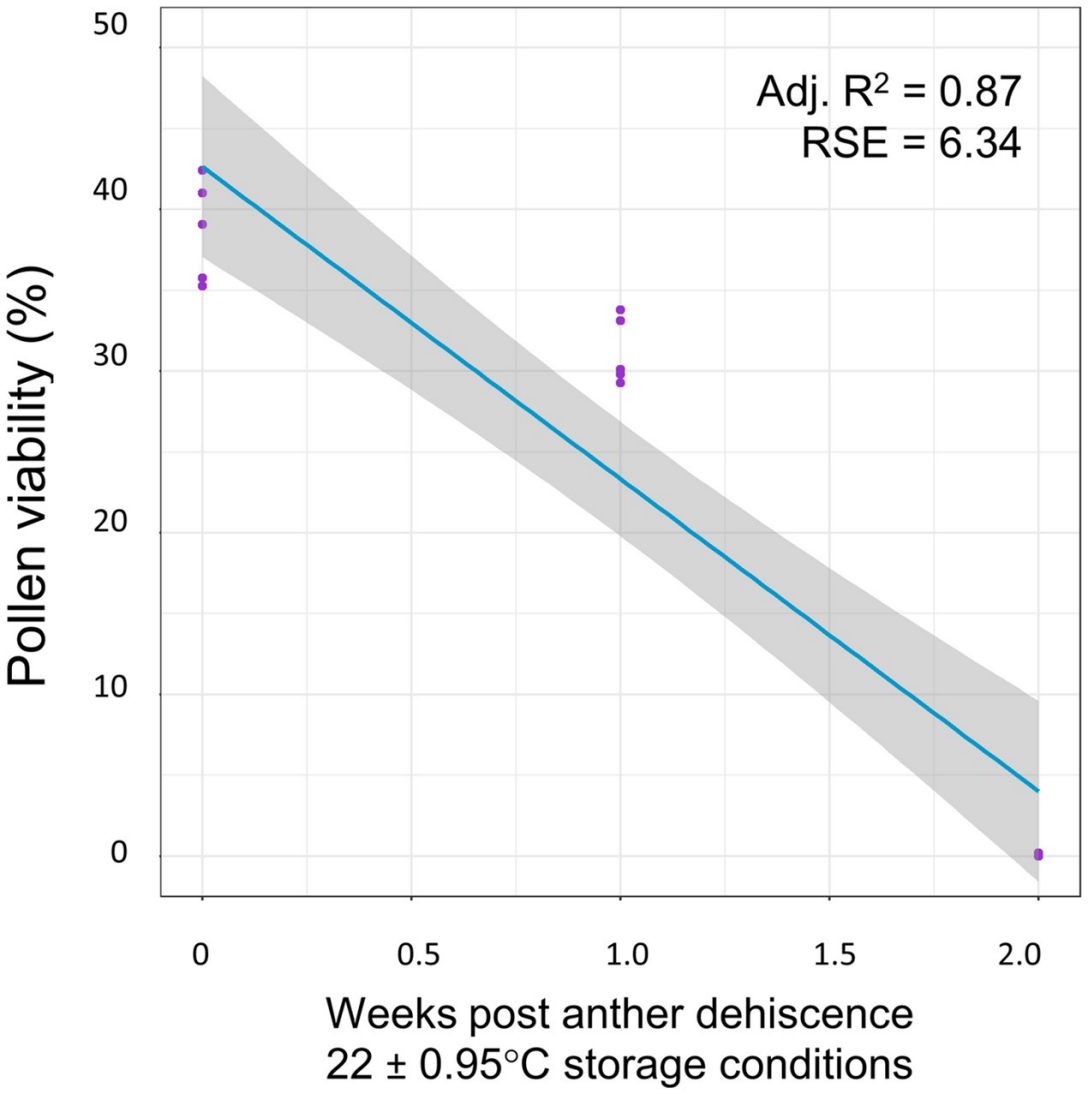
Pollen grain viability under room temperature conditions. The linear relationship between weeks post anther dehiscence and the viability of pools of pollen grains stored under room temperature conditions (22 ± 0.95°C).

Pollen stored under room temperature conditions (22 ± 0.95°C) quickly declined in viability, reaching 0% germination two weeks after anther dehiscence. The linear model fitted to predict pollen viability when stored under room temperature conditions showed a moderate model fit (Adj. R^2^ = 0.87, RSE = 6.34, df = 13, F = 92.67, p < 0.001; Fig 3). Pollen stored in a freezer (-4°C) showed no germination regardless of storage time, after testing *in vitro* germination at both one and two weeks and seeing no pollen tube growth we discontinued collecting data on viability of frozen pollen.

**Fig 4:**
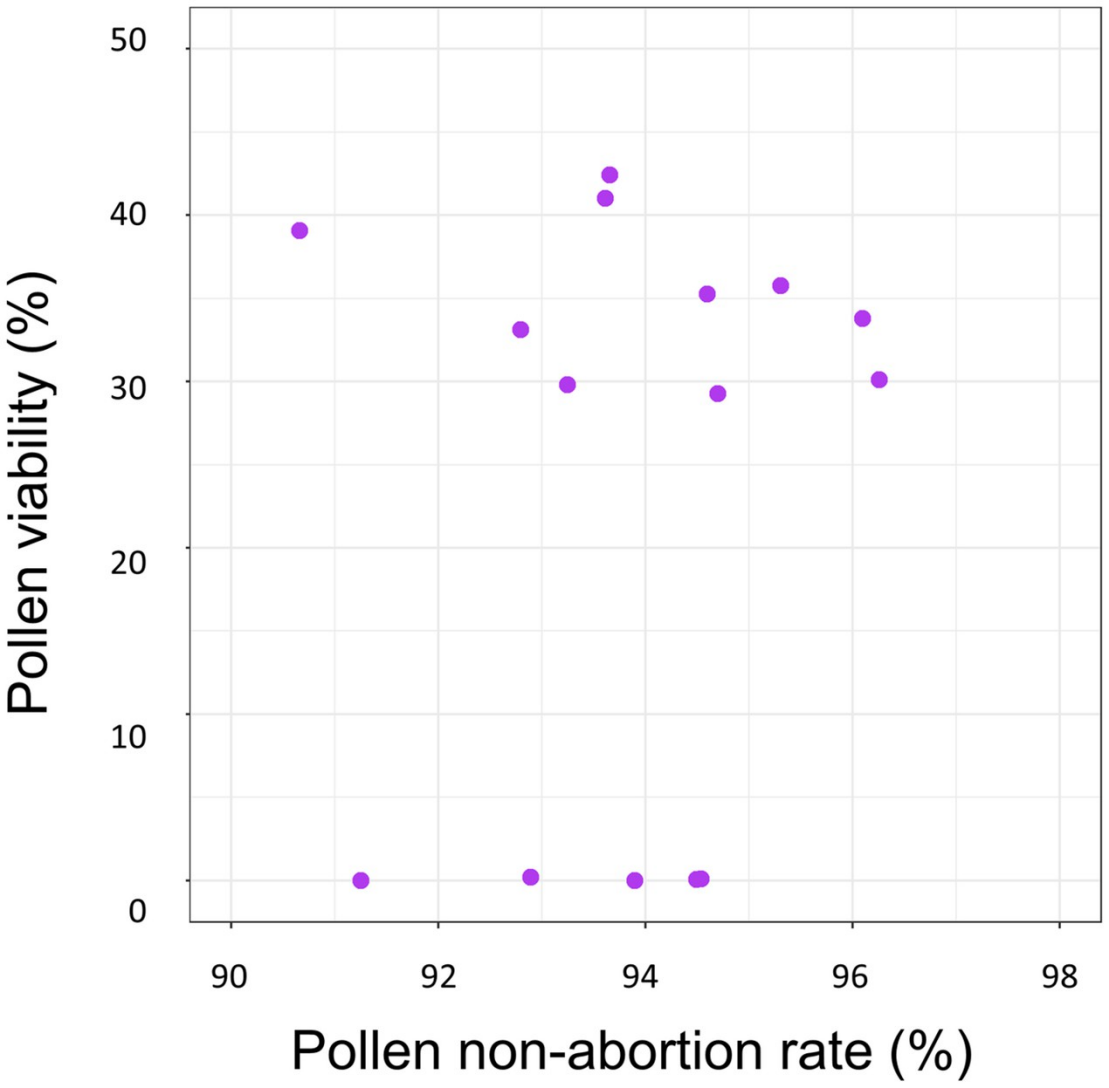
Pollen grain non-abortion rates do not predict viability. The relationship between viability and the abortion rate of pollen grains from a single sample. The associated linear model has insufficient model fit (Adj. R^2^ = -0.06, RSE = 17.93); Wilcoxon signed rank analysis determined that the two characteristics were independent, showing no detectable relationship (v = 120, p < 0.001).

Pollen non-abortion rates did not significantly predict pollen viability using a linear model, when both traits were measured for a single sample (Adj. R^2^ = -0.06, RSE = 17.93, df = 13, F = 0.23, p = 0.64). Moreover, paired measures of pollen viability and non-abortion rates showed no detectable association using non-parametric approaches to analysis (Wilcoxon signed rank test: v = 120, p < 0.001), further demonstrating that pollen viability and abortion rates are independent measures of male gametophytic fitness and do not express any dependent linear relationship (Fig 4).

## Discussion

Our work has demonstrated that pollen viability and non-abortion rates, two differential measures of male gametophytic fitness, are independent of each other and show no correlation in *C*. *sativa*. This coincides with previous *C*. *sativa* research by Zottini *et al*., wherein they also found no relationship between these characteristics [55]. This may be due, in part, to contrasting sensitivity to storage conditions and the external environment; *in vitro* germination requires rehydration and pollen tube growth, a complex biochemical and physiological process [23]. Comparatively, measures of pollen non-abortion rates may rely on pollen senescence, a simpler process that may be less sensitive to storage conditions and the external environment [59]. Pollen viability appeared to be more sensitive to environmental degradation than non-abortion rates, as it quickly declined within two weeks of anther dehiscence, and pollen stored in the freezer (-4°C) did not germinate regardless of storage time, potentially as a result of insufficient dehydration. Interestingly, fresh pollen incubated in a germination media directly from dehiscent anthers did not exceed 42.4% germination, with an average of 38.7% at this time point, similar to the results obtained by Gaudet *et al*., who developed the media recipe and found that fresh pollen germinated at rates varying between 30–50% depending on phenological behaviour [57]. This could imply that viability is generally low in *C*. *sativa*, or that the germination media could be further optimized to reach germination rates exceeding 50%. Previous research investigating pollen viability in other species has demonstrated that *in vitro* germination rates can exceed 80% through optimization of the content of sucrose, polyethylene glycol, and other growth inducing additives [39], while others have shown that relative humidity can strongly influence maximum germination rates under standardized conditions [60]. More recently, optimization of *in vitro* germination in another crop (*Pheonix dactylifera*) has also failed to exceed a threshold of 60%, implying that *C*. *sativa* is not the only species that may struggle to germinate in a liquid media [61]. Bearing this in mind, its probable that quantitative measures of viability in a germination media do not reflect real life fertility rates, and therefore may only be used as a relative indicator of reproductive performance.

Pollen non-abortion rates under room temperature conditions reached a maximum of 95.8%, and averaged 93.3% for freshly dehiscent pollen. This may imply that most pollen produced by male *Cannabis* plants are capable of engaging in reproduction under optimal conditions, and very few pollen grains do not contain an intact regenerative nucleus following maturation of the pollen sacs. The proportion of non-aborted pollen under room temperature conditions averaged 68.8% 12-weeks after anther dehiscence, exceeding the approximate length of the *Cannabis* life cycle. This result may demonstrate that pollen senescence is a process that could be manipulated to preserve pollen samples for extended periods of time. This is verified by the low abortion rates of pollen grains stored in the freezer for 96 weeks post anther dehiscence, which averaged 63.1% of pollen grains containing an intact regenerative nucleus, with a maximum of 69.3% and a minimum of 58.8%. The linear regressions used to model the relationship between storage time and abortion rates predicted that all pollen grains would degrade at 38.3 weeks under room temperature conditions (Adj. R^2^ = 0.8903), and 261.5 weeks under freezer conditions (Adj. R^2^ = 0.8991), suggesting that long-term storage of pollen samples for genotyping is feasible. Furthermore, any pollen that escapes into growth rooms may maintain an intact gamete, long after the crop has been harvested. However, the steady decline in viability that occurred under both experimental conditions could prevent use of stored pollen samples for breeding programs, though it may be possible to extend the decline in viability if storage conditions and the germination media were further optimized.

Through development and testing of these methods for characterizing male gametophytic fitness in *C*. *sativa* we have shown that the pollen of this species maintains an intact gamete for extended periods of time but do not maintain viability under these conditions beyond two weeks after anther dehiscence. Though these results are promising, some limitations to our work should be acknowledged. The long-term maintenance of intact gametes documented in this experiment does not coincide with previous research on this species [56], potentially as a result of differences in methodology or genotypes used. The use of early flowering males limits our ability to investigate if phenological behaviour influences viability, as documented by Gaudet *et al*. [57]. Future work on this topic should include multiple genotypes and pollen collected at different developmental checkpoints to investigate how these factors influence measures of male gametophytic fitness.

## Supporting information

S1 Data Experiment data. All data underlying the findings is available in the attached file.
(XLSX)
